# Supplementary material for: Ensuring privacy and confidentiality of cloud data: A comparative analysis of diverse cryptographic solutions based on run time trend
Source: PLoS One. 2023 Sep 7;18(9):e0290831. doi: 10.1371/journal.pone.0290831 (PMC10484459; doi:10.1371/journal.pone.0290831)
Supplement: Attachment — Submitted filename: ADDRESSED COMMENTS.docx [file pone.0290831.s001.docx]

MANUSCRIPT ID: **PONE-D-23-09341**

TITLE: **ENSURING PRIVACY AND CONFIDENTIALITY OF CLOUD DATA: A COMPARATIVE ANALYSIS OF DIVERSE CRYPTOGRAPHIC SOLUTIONS BASED ON RUN TIME TREND**

Dear Reviewer and Editor,

The authors wish to express their profound gratitude to the Editor and Reviewers for their time in evaluating our manuscript and for suggesting such valuable comments to help raise the quality of the paper to a standard that can be published in Plos one. Given this, an attempt has been made to address all queries and suggestions as much as possible.

The comments of the Reviewers have been written in black, followed by the Authors’ responses in yellow and pink color indicating how the changes have been incorporated.

We trust we have met the expectations of the Editor and Reviewers.

**Author detailed response:**

Reviewer One

All the comments address for reviewer 1 are highlighted in YELLOW

| **S/N** | **COMMENT** | **RESOLUTION/REBUTTAL** | **PAGE** |
| --- | --- | --- | --- |
| 1. | The way of writing is not good | The authors have rewritten the entire paper. The changes resulted in a significant change to the paper.  The authors have also used grammarly to ensure that simple grammatical errors, mistypes, or any other language related deficiencies are corrected. | The entire work |
| 2. | The authors have failed to analyse the performance of the proposed work with existing works mathematically. | This paper seeks to study and comparatively analyze the chosen algorithms to ascertain a comprehensive results of the best performing methods in terms of encryption and decryption time, memory usage, and throughput. All the algorithms are published with detailed mathematical expressions. References to these papers have been provided in appropriate sections of the paper. | NA |
| 3. | Novelty of the work is not good. | The novelty of the work has been reworked in section section 2. | Page 4 |
| 4. | The results are not promising results. | The results section of the work has been re-written and chronologically arranged. | Page 9-16 |
| 5. | Finally, the manuscript contains many grammatical and typographical errors. For example, in abstract, check "This, therefore, raises the need to compare". Also, check the statement "Apica, which is a network traffic control entity also suggested". So strong English proof reading is a must. | The authors have rewritten the entire paper.  The authors have also used grammarly to ensure that simple grammatical errors, mistypes, or any other language related deficiencies are corrected.  All the errors identified by the reviewer and others have been corrected. | Page 1-25 |

Reviewer two

All the comments address for reviewer 1 are highlighted in PINK

| **S/N** | **COMMENT** | **RESOLUTION/REBUTTAL** | **PAGE** |
| --- | --- | --- | --- |
|  | Rrelated work section should be rewritten to improve the readability. | The related work section has been rewritten. | Page 4 |
|  | Authors please explain Calculate ∅() = ( + 1). ( − 1). ( +) . ( − 1) in section 3.2.3 | The reformatting of the work has resulted in the removal of this section.  The authors would be glad of the reviewer could review the paper in its current form. | This part has been removed from the work |
|  | There is no security analysis available in this paper. | The objective of this paper is to compare the performance metrics of the five algorithms based on encryption time trend, Decryption time trend, throughput and memory usage |  |
|  | The authors should clearly describe the novelty of the proposed work. The objectives (privacy & Confidentiality) are not clearly met and justified in this paper through proper security analysis. | The authors have clearly stated the novelty of the work | Page 3 |
