# Supplementary material for: Ensuring privacy and confidentiality of cloud data: A comparative analysis of diverse cryptographic solutions based on run time trend
Source: PLoS One. 2023 Sep 7;18(9):e0290831. doi: 10.1371/journal.pone.0290831 (PMC10484459; doi:10.1371/journal.pone.0290831)
Supplement: Attachment — Submitted filename: REVIEWER 2.docx [file pone.0290831.s002.docx]

REVIEWER 2

MANUSCRIPT ID: PONE-D-23-09341

TITLE: ENSURING PRIVACY AND CONFIDENTIALITY OF CLOUD DATA: A COMPARATIVE ANALYSIS OF DIVERSE CRYPTOGRAPHIC SOLUTIONS BASED ON RUN TIME TREND

Dear Editor and Reviewers,

The authors would like to convey their heartfelt appreciation to the Editor and Reviewers for taking the time to evaluate our submission and for making such insightful comments that could enhance the quality of the work to a level suitable for publication in PLOS ONE. Given this, every effort has been taken to address as many questions and ideas as possible.

The Reviewers' criticisms are written in black, followed by the Authors' answers in green.

We believe we have met the Editor's and Reviewers' expectations.

**Author detailed response:**

Reviewer

All the comments from the reviewer are written in red in the response sheet.

The responses to the reviewer’s comments are highlighted green under resolution.

| **S/N** | **COMMENT** | **RESOLUTION/REBUTTAL** | **PAGE** |
| --- | --- | --- | --- |
| 1. | Authors should assign chapter numbers carefully. There are several mistakes in the numbering throughout the paper. For example, please check the conclusion section number, as well as section 3.4.4, which is mistakenly labeled as 3.3.5. | The manuscript has been revised |  |
| 2. | The overall organization of the article is difficult to understand, and it lacks continuity. The authors should revise the structure and flow of the paper to ensure a logical and coherent progression of ideas. | The manuscript has been well structured now |  |
| 3 | Please check equation numbers carefully. There is an instance where equation number 13 is represented twice. Ensure that all equations are properly numbered and referenced. | The equation numbers have been well arranged now |  |
| 4 | The flow of the article is lacking in explanations about tables and figures. Make sure to provide clear and sufficient explanations for each table and figure to help readers understand their relevance and implications. | The Figures and Table have been given clear explanations |  |
| 5 | The manuscript contains numerous grammatical and typographical errors. The authors are advised to thoroughly proofread the paper and consider professional English proofreading services to improve the language quality. | The grammatical errors have been corrected |  |
| 6 | The novelty of the work needs to be more explicitly described. The authors should clearly state the unique contributions of their research and highlight how it differentiates from previous works in the field. | The problem statement for this manuscript has been spelt out. Based on this the novelty has been clearly stated in the abstract. | 1 |
